# Genotypic diversity of *Streptococcus suis* and the *S. suis*-like bacterium *Streptococcus ruminantium* in ruminants

**DOI:** 10.1186/s13567-019-0708-1

**Published:** 2019-11-14

**Authors:** Masatoshi Okura, Fumito Maruyama, Atsushi Ota, Takeshi Tanaka, Yohei Matoba, Aya Osawa, Sayed Mushtaq Sadaat, Makoto Osaki, Atsushi Toyoda, Yoshitoshi Ogura, Tetsuya Hayashi, Daisuke Takamatsu

**Affiliations:** 1 Division of Bacterial and Parasitic Diseases, National Institute of Animal Health, National Agriculture and Food Research Organization, Tsukuba, Japan; 2 Department of Microbiology, Graduate School of Medicine, Kyoto University, Kyoto, Japan; 3 Scientific and Technological Bioresource Nucleus, Universidad de La Frontera, Temuco, Chile; 4 Nairiku Meat Inspection Center, Yamagata Prefectural Government, Yamagata, Japan; 5 Murayama Public Health Center, Yamagata Prefectural Government, Yamagata, Japan; 6 Matsumoto Livestock Hygiene Service Center, Nagano Prefectural Government, Matsumoto, Japan; 7 Ministry of Agriculture, Irrigation and Livestock, Animal Health Directorate, Central Veterinary Diagnostic and Research Laboratory, Kabul, Afghanistan; 8 Department of Genomics and Evolutionary Biology, National Institute of Genetics, Shizuoka, Japan; 9 Advanced Genomics Center, National Institute of Genetics, Shizuoka, Japan; 10 Department of Bacteriology, Faculty of Medical Sciences, Kyushu University, Fukuoka, Japan; 11 United Graduate School of Veterinary Sciences, Gifu University, Gifu, Japan

## Abstract

Although *Streptococcus suis* has attracted public attention as a major swine and human pathogen, this bacterium has also been isolated from other animals, including ruminants. However, recent taxonomic studies revealed the existence of other species that were previously identified as *S. suis*, and some of these isolates were reclassified as the novel species *Streptococcus ruminantium*. In Japan, biochemically identified *S. suis* is frequently isolated from diseased ruminants; however, such isolates have not yet been identified accurately, and their aetiological importance in ruminants is unclear. Therefore, to understand the importance of *S. suis* and *S. suis*-like bacteria in ruminants, we reclassified *S. suis* isolates from ruminants according to the updated classification and investigated their genetic diversity. Although both *S. suis* and *S. ruminantium* were isolated from healthy and diseased ruminants, most of the isolates from diseased animals were *S. ruminantium*, implying that *S*. *ruminantium* is more likely to be associated with ruminant disease than *S. suis*. However, the ruminant *S. suis* and *S. ruminantium* isolates from diseased animals were classified into diverse genotypes rather than belonging to certain clonal groups. Genome sequence analysis of 20 *S*. *ruminantium* isolates provided information about the antibiotic resistance, potential virulence, and serological diversity of this species. We further developed an *S*. *ruminantium*-specific PCR assay to aid in the identification of this bacterium. The information obtained and the method established in this study will contribute to the accurate diagnosis of ruminant streptococcal infections.

## Introduction

*Streptococcus suis* is a Gram-positive bacterium that is isolated from the upper respiratory tract of pigs [1]. *S. suis* is recognized as an emerging zoonotic pathogen that has been linked to various diseases, including septicaemia, meningitis, and endocarditis, in both swine and humans [2–4]. Multilocus sequence typing (MLST) has been the most widely used genotyping tool for *S. suis* [3, 5]. As of March 2019, 1170 sequence types (STs) had been described [6], and the zoonotic isolates were grouped into several clonal complexes (CCs) [3, 7]. *S. suis* has traditionally been classified into 35 serotypes on the basis of antigenic differences in its capsular polysaccharides [7]. Among these serotypes, serotype 2 is most commonly associated with systemic infections in pigs and humans [3]. These genotypic and serological data suggest that *S. suis* human infections are mainly caused by the strains present in pigs. However, *S. suis* infections also occur in other animals, including ruminants [2, 8, 9]. Due to the limited genotypic and serological data available for the isolates from these animals, the epidemiological relationship between zoonotic isolates and isolates from other animals remains unclear.

Although *S. suis* is considered to be a species with genetically and serologically diverse strains, recent taxonomic analysis has led to the reclassification of several *S. suis* serotype reference strains [7, 10–12]. Currently, *S. suis* reference strains of serotypes 32 and 34 are regarded as *Streptococcus orisratti* [10], and those of serotypes 20, 22 and 26 are regarded as *Streptococcus parasuis* [11]. In 2017, Tohya et al. [12] proposed the reclassification of the serotype 33 reference strain as the new species *Streptococcus ruminantium*. Additionally, several *S. suis*-like isolates that were previously identified as *S. suis* but are not taxonomically considered *S. suis* have been found [7]. These *S. suis*-like strains are difficult to distinguish from *S. suis* by biochemical tests [7]; however, they can be detected by PCR amplification of the glutamate dehydrogenase gene (*gdh*-PCR) [13], which is widely used as a detection method for *S. suis* [7, 14] since the *gdh*-PCR approach was designed to detect 35 serotype reference strains, including six reference strains (serotypes 20, 22, 26, and 32–34) that are currently regarded as different species [13]. As such, in many clinical cases, *S. suis*-like strains may be misidentified as *S. suis* in diagnostic laboratories. To accurately discriminate between *S. suis* and *S. suis*-like bacteria, Ishida et al. [14] developed a PCR strategy targeting a DNA repair protein gene (*recN*-PCR), in which the primers designed to discriminate the serotype reference strains of authentic *S. suis* from the six reference strains reclassified as other species. This *recN*-PCR method is currently used in diagnostic laboratories for *S. suis* identification [15, 16].

Among the *S. suis*-like strains, *S. ruminantium* has been isolated from cattle with endocarditis and a lamb with arthritis [12, 17], suggesting that *S. ruminantium* is associated with ruminant disease. Isolation of *S. suis* from diseased ruminants has also been reported [18–22]; however, many of these reports were published prior to the proposal of *S. ruminantium* as a species. Given that some of these isolates may have been misidentified, the aetiological importance of *S. suis* and *S. suis*-like bacteria, including *S. ruminantium*, in ruminants remains unknown. Correct identification of these streptococcal isolates is crucial for the precise diagnosis of streptococcal disease and an accurate understanding of the role of these bacteria in ruminants.

In the diagnostic service of the National Institute of Animal Health in Japan (NIAH-Japan), we have encountered many “*S. suis*” isolates from ruminants that were identified by biochemical tests and/or *gdh*-PCR. In this study, to understand the aetiological importance of *S. suis* and *S. suis*-like bacteria in ruminants, these “*S. suis*” isolates were characterized on the basis of the recent classification; a PCR strategy for detecting *S. ruminantium* was developed; and the presence of these bacteria in the tonsils of healthy cattle was investigated. Reidentified and newly isolated *S. suis* and *S. ruminantium* strains were further genotyped by MLST or pulsed-field gel electrophoresis (PFGE) analysis as well as typing of the capsular polysaccharide synthesis genes (*cps*), where the *cps* types were numbered according to the expected serotypes (e.g., serotype 3 for *cps* type 3); however, serotypes 1 and 1/2 cannot be distinguished from serotypes 14 and 2, respectively [23]. Furthermore, 20 of the *S*. *ruminantium* isolates were subjected to genome sequence analysis to gain insights into the pathogenicity and antibiotic resistance of *S. ruminantium* strains.

## Materials and methods

### Bacterial strains

Sixty-four streptococcal isolates were collected between 1992 and 2017 in our laboratory as part of the diagnostic services provided by NIAH-Japan (Table 1 and Additional file 1). Fifty-six of the strains were isolated from cattle (*n* = 53), sheep (*n* = 2), and a goat (*n* = 1) that were either diseased or dead, although 18 of the strains were isolated from sites that were likely unrelated to the disease or where information on clinical signs was lacking. The remaining isolates came from healthy cattle (*n* = 7) and a milk-feeding robot for calves (*n* = 1). All of the ruminant isolates were collected from different animals, although 12 of them and one environmental isolate came from the same farm (Additional file 1). These 64 isolates had been previously identified as *S. suis* by *gdh*-PCR [13] and the API 20 Strep system (BioMérieux, France) or the Rapid ID 32 Strep system (BioMérieux).

**Table 1:** Information for the 64 *gdh*-PCR positive *Streptococcus* spp. isolates from ruminants collected by the diagnostic service of NIAH-Japan

Source	Clinical history	Isolation site	No. of strains				
			Total	Strains that were isolated with other agents^a^	*S. suis*^b^	*S. ruminantium*^b^	*S. parasuis*^b^
Cattle	Meningitis	Brain	1	0	1	0	0
	Astasia	Cerebrospinal fluid	1	0	1	0	0
	Astasia and pneumonia	Cerebrospinal fluid	1	1	1	0	0
	Astasia, respiratory disease and endocarditis	Heart	1	0	1	0	0
	Endocarditis	Heart	6	1	0	6	0
	Arthritis	Pus in the articular cavity	1	0	0	1	0
	Torticollis and respiratory disease	Abscess in tympanic cavity	1	0	0	1	0
	Pneumonia or respiratory diseases	Pulmonary abscess	3	1	0	3	0
		Lung	20	12	0	20	0
	Death	Liver abscess	1	0	0	1	0
		Pulmonary abscess	1	1	0	1	0
	Unclear or unknown^c^	Oral cavity	5	–	2	3	0
		Tonsil	5	–	0	5	0
		Lung	6	–	0	6	0
	Healthy	Oral cavity	4	–	2	2	0
		Nasal cavity	1	–	0	0	1
		Tonsil	1	–	1	0	0
		Lung	1	–	0	1	0
Sheep	Pneumonia	Pulmonary abscess	1	0	0	1	0
	Unclear or unknown^c^	Tonsil	1	–	0	1	0
Goat	Unclear or unknown^c^	Lung	1	–	0	1	0
Milk feeding robot	–	Papillary area	1	–	0	1	0
Total			64	16	9	54	1

^a^Only recorded cases are indicated (Additional file 1).

^b^Identified based on 16S rRNA gene sequencing.

^c^Isolated from diseased animals at sites that were unlikely to be related to the disease or were unknown due to a lack of information on diseases and clinical signs.

An additional 55 streptococcal isolates were collected from the tonsils of 110 cattle during a meat inspection at the Yamagata Nairiku Meat Inspection Center in Japan in 2012 (Additional file 2). The strains were isolated from aseptically dissected tonsillar samples, from which the surface stamps were streaked on Colistin-oxolinic acid blood agar [Columbia agar (Becton–Dickinson, Sparks, MD, USA) supplemented with 5% defibrinated sheep blood and Streptococcus-selective supplement (10 mg/L of colistin sulphate and 5 mg/L of oxolinic acid; Oxoid, UK)] [24]. After incubation at 37 °C for 1–2 days in a candle jar, up to 8 α-haemolytic colonies from each tonsillar sample were selected for Gram staining and catalase and oxidase tests. Gram-positive, catalase-negative, and oxidase-negative cocci were further characterized using the API 20 Strep system and *gdh*-PCR [13]. The isolates identified as *S. suis* by both the API 20 Strep system and *gdh*-PCR were used in this study.

### DNA extraction, PCR, and amplicon sequencing

All the isolates were cultured overnight on Todd-Hewitt (TH) agar (Becton–Dickinson) at 37 °C under aerobic conditions with 5% CO_2_. InstaGene Matrix (Bio-Rad, Hercules, CA, USA) was used to extract bacterial DNA according to the manufacturer’s instructions. All of the PCR assays were performed using TaKaRa Ex *Taq* polymerase (Takara Bio Inc., Kusatsu, Japan) and QIAGEN Multiplex Master PCR Mix (Qiagen, Hilden, Germany), according to the manufacturers’ instructions. For sequencing, the amplified PCR products were purified using the QIAquick PCR purification kit (Qiagen) following the manufacturer’s instructions and sequenced with a 3130*xl* Genetic Analyzer (Applied Biosystems, Foster City, CA, USA) using a BigDye Terminator v3.1 cycle sequencing kit (Applied Biosystems). Sequence assembly was performed using SEQUENCHER 5.4 (Gene Codes Corp., Ann Arbor, MI, USA).

### PCR for *S. suis* identification and typing

All the isolates (*n* = 119) were subjected to *gdh*-PCR as previously described [13]. For the identification of authentic *S. suis*, *recN*-PCR was performed as previously described [14]. The 119 isolates were also analysed by *cps*-typing, which is a two-step multiplex PCR method used for the typing of the *cps* gene cluster of *S. suis*, as previously described [23]. Moreover, we performed MLST according to published protocols using clinical isolates of authentic *S. suis* [5]. The STs were determined using the *S. suis* MLST database. Novel alleles and STs were assigned through the submission of the data to the database.

### Sequence analysis of 16S rRNA and superoxide dismutase (*sodA*) genes

The 16S rRNA gene fragments (> 1.5 kbp) of all isolates were amplified and sequenced as previously described [25]. Pairwise similarities between the 16S rRNA gene sequences of the isolates used in this study and the type strain of each species were calculated using the EzBioCloud server [26] and the web Basic Local Alignment Search Tool (BLAST) of the National Center for Biotechnology Information (NCBI). In this study, 98.65 % sequence similarity was used as the threshold for differentiating two species [27].

With regard to the isolates identified as *S. ruminantium* by 16S rRNA gene sequencing, a partial sequencing analysis of the superoxide dismutase gene (*sodA*) was conducted as an additional assay for species identification to identify more precisely, referring to a previous report [12]. The amplification and sequencing of *sodA* were performed as previously described [28]. BLAST analysis was conducted to calculate the similarities between the tested isolates and the *S. ruminantium* type strain GUT-187^T^ (GenBank accession no. LC195049).

### PCR for the discrimination of *S. ruminantium* from *S. suis*

Primers were designed as follows: the 16S rRNA gene sequences from 35 *S. suis* serotype reference strains (serotypes 1–34 and 1/2) and *S. ruminantium* GUT-187^T^ and DAT741 were aligned by MAFFT FFT-NS-i v7.215 [29] with the default parameters. The alignment profile was then manually searched for the distinctive regions of *S. ruminantium* GUT-187^T^, DAT741 and EA1832.92 (serotype 33). The specificity of the potential primer regions was confirmed using the Ribosomal Database Project’s (RDP) Probe Match algorithm (Release 11, Update 5) [30]. Accordingly, the sequences of the forward and reverse primers were 5′-GCAAGTGGAACGCAACTTTTCA-3′ and 5′-CTATGTATCGTTGCCTTGGTAG-3′, respectively. The 16S rRNA gene sequences of the *S. suis* strains used for this analysis were obtained from their draft genome sequences [31]. PCR was carried out using TaKaRa Ex *Taq* polymerase (Takara Bio Inc.) under the following conditions: 95 °C for 2 min, followed by 30 cycles of 95 °C for 20 s, 60 °C for 10 s, and 72 °C for 20 s.

To evaluate primer specificity, the PCR assay was tested with 12 type strains of *Streptococcus* spp., three ruminant-derived *S. suis* serotype reference strains, two *S. orisratti* strains (*S. suis* serotype 32 and 34 reference strains), ten streptococcal field isolates from ruminants (Additional file 3), and the 119 isolates analysed in this study. The analytical sensitivity of the PCR assay, corresponding to the minimum number of bacterial cells that are detected by the assay, was determined using *S. ruminantium* DAT741. In brief, the strain was grown to an optical density at 600 nm of 0.1 in TH broth, at which point the genomic DNA was extracted from 100 µL of a bacterial suspension with 100 μL of InstaGene Matrix (Bio-Rad). Ten-fold serial dilutions of the extracted DNA were used for the PCR assay. The number of colony-forming units (CFUs) of the above bacterial suspensions was also determined by plating serial dilutions on TH agar, followed by incubation overnight at 37 °C under aerobic conditions with 5% CO_2_.

### PFGE

PFGE was performed according to the procedures described previously [32] with slight modifications. Briefly, bacterial cells were harvested from brain heart infusion (BHI) agar (Becton–Dickinson), washed with Tris-saline buffer (10 mM Tris–HCl [pH 8.0], 1 M NaCl), and suspended in EDTA–sarcosine buffer (6 mM Tris–HCl, 1 mM NaCl, 100 mM EDTA, 1% sodium *N*-lauroylsarcosine; pH 7.6). The suspension was mixed with an equal volume of 1.0% SeaKem Gold agarose (Lonza Rockland, Rockland, ME, USA) in EDTA–sarcosine buffer, and the mixture was solidified in a 0.7-mm sample plug caster (Bio-Rad). The sample plugs were then incubated in lysis buffer (0.5 M EDTA [pH 8.0], 2.5 mg/mL lysozyme, 10 U/mL mutanolysin) for 3 h at 37 °C, followed by proteinase K solution (0.5 M EDTA [pH 8.0], 1% sodium *N*-lauroylsarcosine, 1 mg/mL proteinase K) for 18 h at 50 °C. The samples were next treated twice with 1 mM Pefabloc SC (Roche Applied Science, Basel, Switzerland) in Tris–EDTA (TE) buffer (10 mM Tris–HCl, 1 mM EDTA: pH 8.0) for 30 min at 50 °C and washed three times with TE at room temperature. The genomic DNA in each plug was then digested with 20 U of SmaI (Takara Bio) for 18 h at 30 °C and separated on a 1.0% SeaKem Gold agarose gel in Tris–borate–EDTA buffer (44.5 mM Tris, 44.5 mM boric acid, 1 mM EDTA: pH 8.0) supplemented with 50 µM thiourea using a CHEF-DR II System (Bio-Rad Laboratories). The electrophoresis conditions were as follows: 5.5 V/cm with pulse times of 2–10 s for 13 h and 20–25 s for 6 h at 14 °C. The PFGE patterns present from 48.5 to 630.5 kbp were analysed on the basis of the Dice-predicted similarity of two patterns, and the unweighted pair group method with average linkage clustering was used to construct the corresponding dendrogram at a setting of 1.0% position tolerance using GelJ v2.0 [33].

### Whole-genome sequencing, annotation, and pangenome analysis

Among the 76 isolates, 10 isolates from diseased sites (three from the heart, one from a liver abscess, one from a pulmonary abscess, five from the lungs) and 10 isolates from the tonsils or oral cavities were selected for whole-genome sequencing in this study (Additional file 4). For these analyses, the DNeasy Blood and Tissue Kit (QIAGEN) was used for the extraction of genomic DNA; genomic libraries were constructed using the Nextera XT DNA Library Preparation kit (Illumina, Inc., San Diego, CA, USA); and genome sequence data were obtained using an Illumina HiSeq 2500 sequencer (Illumina). Adapter and low-quality sequences were trimmed from the generated read sequences using FastQC and Trimmomatic [34]; the resulting sequences were assembled using Velvet v1.2.08 [35]. *S. ruminantium* DAT741 from a diseased cow with endocarditis was also sequenced on the PacBio RSII platform (Pacific BioSciences Inc., Menlo Park, CA, USA) at Macrogen Japan Corp. (Kyoto, Japan) to determine its complete genome sequence. De novo genome assembly was performed via the Hierarchical Genome Assembly Process (HGAP3) with SMRT Portal v2.3.0, and annotation of the genome assemblies was executed with Prokka v1.12 (default setting) [36]. The circular map of the *S. ruminantium* DAT741 chromosome was visualized using in silico MolecularCloning software (In Silico Biology, Inc., Japan). Thereafter, the pangenome was analysed using Roary v3.11.2 (with default parameters) [37]. Genes related to antibiotic resistance were searched using the Resistance Gene Identifier in the Comprehensive Antibiotic Resistance Database (CARD) [38]. Contigs including antibiotic resistance genes were compared to all integrative conjugative element (ICE) sequences in ICEberg v2.0 [39] using web nucleotide BLAST.

### Antibiotic susceptibility testing

The minimal inhibitory concentrations (MICs) of tetracycline, erythromycin, chloramphenicol, kanamycin, streptomycin, amoxicillin, ampicillin, and benzylpenicillin were measured on Mueller–Hinton agar (Oxoid) containing defibrinated 5% sheep blood using Etest strips (BioMérieux) according to the manufacturers’ instructions.

### Identification and typing of the putative *cps* gene clusters of 21 *S. ruminantium* strains

To identify putative *cps* gene clusters, contigs that contained genes similar to those of the *cps* gene cluster and its flanking genes *orfX,* encoding a hypothetical protein, and *glf*, encoding the UDP-galactopyranose mutase, of EA1832.92 (GenBank accession no. AB737837) were isolated from the pangenome data using Roary. Gaps between contigs were amplified by PCR, and their sizes were compared to unigenes with no gaps. The 21 putative *cps* gene clusters were classified according to their genetic organization based on the pangenome data using Roary. The Artemis Comparison Tool (ACT) [40] was used to visualize the comparison data between two sequences using BLASTN (bit-scores above 50 and E-values lower than 1e–8 were displayed).

Primers were then designed against the characteristic regions of each identified *cps* gene cluster type to classify the *cps* types of the *S. ruminantium* strains. The target *cps* genes, primer sequences, and predicted size(s) of each PCR product are shown in Additional file 5. All of the PCR assays were carried out using TaKaRa Ex *Taq* polymerase (Takara Bio Inc.) under the following conditions: 95 °C for 2 min followed by 30 cycles of 95 °C for 20 s, 60 °C for 10 s, and 72 °C for 40 s.

### Data availability

All of the sequences determined in this study were deposited in the DDBJ/ENA/GenBank databases under accession numbers LC316845-LC316868, LC316870-LC3169000, LC316903-LC316941, LC377185-LC377187, LC337291-LC337338, LC337341-LC337368, BCFA01000001-BCFA01000063, BCFD01000001-BCFD01000049, BCFE01000001-BCFE01000040, BCFF01000001-BCFF01000047, BCFB01000001-BCFB01000063, BCEZ01000001-BCEZ01000068, BCFG01000001-BCFG01000058, BCFH01000001-BCFH01000058, BCFI01000001-BCFI01000050, BCFC01000001-BCFC01000061, BCES01000001-BCES01000040, BCEY01000001-BCEY01000040, BCET01000001-BCET01000041, BCEU01000001-BCEU01000040, BCEP01000001-BCEP01000043, BCEV01000001-BCEV01000045, BCEQ 01000001-BCEQ 01000156, BCEW01000001-BCEW01000044, BCER01000001-BCER01000040, BCEX01000001-BCEX01000031, and CP019557 (Additional files 1, 2, 3 and 4).

## Results

### Ruminant “*S. suis”* isolates were frequently reclassified as *S. ruminantium*

The 16S rRNA gene sequence analysis of the ruminant “*S. suis*” isolates collected through the diagnostic service of NIAH-Japan indicated that most of the isolates (55/64) were not *S. suis*, as the pairwise sequence similarities between their respective 16S rRNA gene sequences and that of *S. suis* S735^T^ were less than 98% (Table 1 and Additional file 1). Accordingly, 54 of these isolates were reclassified as *S. ruminantium* based on the pairwise sequence similarity of the 16S rRNA (99.08–100%) and *sodA* (99.77–100%) genes between *S. ruminantium* GUT-187^T^ and the respective isolates. The 16S rRNA gene sequence of the remaining isolate was identical to that of *S. parasuis* STU-286^T^ (Additional file 1).

The 64 analysed *S. ruminantium* isolates were isolated from both healthy cattle and cattle with diseases including pneumonia, respiratory diseases, endocarditis, arthritis, and torticollis (Table 1 and Additional file 1). Similarly, *S. suis* was isolated from four calves with meningitis and astasia and five healthy cattle. Furthermore, these isolates were identified as *S. suis* by *recN*-PCR (Additional file 1).

### PCR to discriminate between *S. ruminantium* and *S. suis*

Next, a PCR strategy was developed to discriminate *S. ruminantium* from *S. suis* and *S. suis*-like bacteria using *S. ruminantium*-specific 16S rRNA primers. The primer design was based on the alignment of the 16S rRNA gene sequences of 35 *S. suis* serotype reference strains and two *S. ruminantium* strains (GUT-187^T^ and DAT741) (Additional file 6). The PCR approach detected the 54 isolates identified in this study as *S. ruminantium* and the serotype 33 reference strain EA1832.92. Furthermore, 37 non-*S. ruminantium* isolates, including 13 *S. suis*, *S. parasuis* and *S. orisratti* strains, were not detected (Table 1 and Additional files 1 and 3). Using the described method with *S. ruminantium* DAT741, the detection limit of this PCR strategy was 2.7 × 10^3^ CFU/mL (Additional file 7).

### Cattle carried *S. suis* and/or *S. ruminantium* in their tonsils

The carrier rates for these bacteria in the tonsils of cattle were investigated. Accordingly, *gdh*-PCR-positive streptococci were isolated from the tonsils of 48 (43.6%) of the tested cattle (*n* = 110; Additional file 2). Further identification by *recN*-PCR and the developed *S. ruminantium*-specific PCR assay with the *gdh*-PCR-positive isolates revealed that 32 and 22 strains belonged to the species *S. suis* and *S. ruminantium*, respectively (Additional file 2). The *S. ruminantium* strains were further confirmed by the analysis of the 16S rRNA and *sodA* genes (Additional file 2).

### The sequence and *cps* types of the cattle *S. suis* clinical isolates were different from those of human and porcine clinical isolates

Given the finding that *S. suis* can cause severe disease in calves, the *S. suis* isolates were also typed according to their *cps* gene clusters. Notably, the four *S. suis* isolates from diseased calves were not classified as *cps* type 2. Instead, two of the isolates were typed as *cps* type 8, one as type 10, and one was untypable (Table 2 and Additional file 8). Furthermore, *cps* typing of the remaining *S. suis* isolates from the oral cavities and tonsils of cattle indicated that *cps* type 8 was the most common (10/37) and that a significant proportion were untypable (23/37; Table 2). MLST classified the four *S. suis* isolates as novel STs (ST999, ST1000, ST1002, and ST1003); these STs did not share at least five alleles each with any STs in the MLST database of *S. suis* (Additional file 8).

**Table 2:** Prevalence and distribution of *cps* types in 41 ruminant *S. suis* isolates

*cps* type^a^	No. of isolates		
	Total, *n* = 41 (%)	Diseased sites^b^, *n* = 4 (%)	Healthy^c^, *n* = 34 (%)
8	12 (29.3)	2 (50.0)	9 (26.4)
9	3 (7.3)	0	3 (8.8)
10	1 (2.4)	1 (25.0)	0
31	1 (2.4)	0	1 (2.9)
UT	24 (58.5)	1 (25.0)	21 (61.8)

UT: untypable.

^a^Types based on *cps*-typing for *S. suis* [23].

^b^Brain, CSF or heart (Table 1 and Additional file 1).

^c^3 of the 64 isolates collected by the diagnostic service of NIAH-Japan (Table 1 and Additional file 1) and 31 of the 32 *S. suis* isolates collected from cattle tonsils (Additional file 2, one isolate came from a cow with intestinal obstruction).

### Classification of *S. ruminantium* isolates by pulsotype

**Figure 1:**
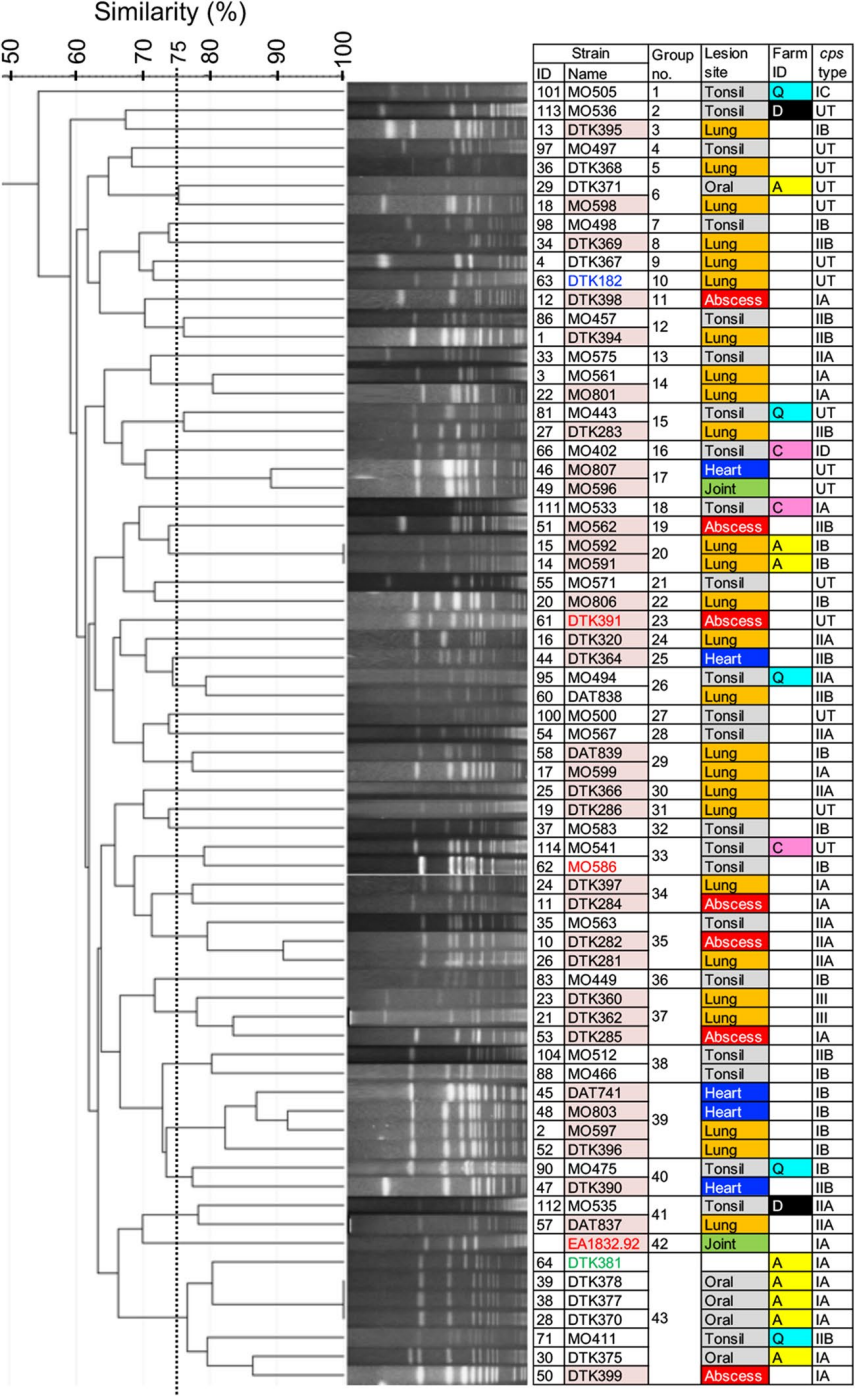
**SmaI-digested PFGE patterns of** ***S. ruminantium.*** The left panel presents the dendrogram based on the PFGE profiles. Some strains, including MO411, MO494, MO535, MO675, and DTK371, seemed to exhibit slightly affected PFGE profiles, probably due to the nuclease degradation of DNA. The right panel presents the strain information (highlighting with a red background corresponds to isolates from the lesions of diseased animals; blue, goat isolate; red, sheep isolates; green, environmental isolate; see Additional files 1 and 2 for more details).

PFGE was performed to evaluate the degree of genetic diversity and clonality of *S. ruminantium* isolates from healthy and diseased ruminants. The PFGE profiles of eight of the tested isolates were not analysed because they showed smearing due to DNA degradation or indigestion. As such, the PFGE patterns of 68 ruminant isolates and EA1832.92 were distinguished, producing 66 different pulsotypes (Figure 1). The 66 pulsotypes were clustered into 43 groups with a 75% similarity cutoff level, meaning that less than three fragment differences existed between the profiles. Many of the clusters (26/43) were composed of a single isolate, whereas 17 clusters contained two to seven isolates. Isolates collected from the same farm were all classified into different groups with the exception of two isolates from group 20 and five from group 43, which were all isolated from Farm A (Figure 1).

### Genome analysis revealed genes present in disease-associated isolates

To investigate whether *S. ruminantium* isolates associated with diseases in ruminants exhibit specific genetic markers, a comparative analysis was performed using the genome sequences of 20 *S. ruminantium* isolates, ten isolates from diseased tissue sites and ten from the tonsils or oral cavity of cattle. The genome sizes of the isolates ranged from 1.98 to 2.29 Mb, and there were 1873–2217 predicted coding sequences (Additional file 4).

Pangenome analysis with Roary identified 4163 clusters of orthologous groups (COGs) in the genes of the 20 *S. ruminantium* isolates and EA1832.92 (Additional file 9). Although there were no COGs that were exclusively conserved in all ten clinical isolates and exclusively absent in seven or more of the other isolates, eleven COGs were specifically present in the clinical isolates from lesions of the heart, abscesses and a joint (Table 3). Furthermore, each of these genes was present in the genome of *S. ruminantium* GUT187^T^, which was isolated from the heart of a cow with endocarditis [41].

**Table 3:** Genes that were characteristic of the *S. ruminantium* isolates from diseased tissue sites

COG ID^a^	Annotation	Conserved domain and/or description on blast hits	Isolation sites										
			Heart			Liver abscess	Pulmonary abscess	Joint	Lung				
			DAT741	DTK364	DTK390	DTK285	DTK284	EA1832.92	MO591	DTK360	DTK397	DTK366	DAT837
fhaB	Iron-regulated surface protein	pfam04650: YSIRK_signal; YSIRK type signal peptide cl27124: IsdB super family; haem uptake protein IsdB	+	+	+	+	+	+	+	+	–	–	+
group_1846	Peptide-binding protein	cd08510: PBP2_Lactococcal_OppA_like; the substrate binding component of an ABC-type lactococcal OppA-like transport system	+	+	+	+	+	+	+	–	–	+	+
*group_669*	Hypothetical protein	Imm70 super family cl21402 Immunity protein 70	+	+	+	+	+	+	–	–	–	–	+
*group_1859*	DNA-binding response regulator	CitB COG2197 DNA-binding response regulator Similar to SalR of *S. suis* SC84 (Amino acid similarity, 86%; coverage, 100%)	+	+	+	+	+	+	–	–	–	–	+
*group_1860*	Histidine kinase	HisKA_3 super family cl26854 Histidine kinase Similar to SalK of *S. suis* SC84 (Amino acid similarity, 84%; coverage, 100%)	+	+	+	+	+	+	–	–	–	–	+
*group_1861*	ABC transporter permease	No conserved domain Blast hit with ABC transporter permeases of several species	+	+	+	+	+	+	–	–	–	–	+
*group_1862*	ABC transporter permease	No conserved domain Blast hit with ABC transporter permeases of several species	+	+	+	+	+	+	–	–	–	–	+
*group_1863*	ABC transporter	ABC_ATPase super family cl25403 ATP-binding cassette transporter nucleotide-binding domain	+	+	+	+	+	+	–	–	–	–	+
*group_1864*	ABC transporter	SunT super family cl26602 ABC-type bacteriocin/lantibiotic exporters	+	+	+	+	+	+	–	–	–	–	+
*group_391*	Lantibiotic-modifying protein	LanC_like super family cl04955 Cyclases involved in the biosynthesis of lantibiotics	+	+	+	+	+	+	–	–	–	–	+
*group_1865*	Columbicin A-like bacteriocin peptide	L_biotic_typeA super family cl04622 Type-A lantibiotic	+	+	+	+	+	+	–	–	–	–	+

COG ID^a^	Annotation	Conserved domain and/or description on blast hits	Tonsils or oral cavity										Heart
			MO402	DTK377	MO430	MO449	MO492	MO498	MO505	MO512	MO533	MO557	GUT187^b^
fhaB	Iron-regulated surface protein	pfam04650: YSIRK_signal; YSIRK type signal peptide cl27124: IsdB super family; haem uptake protein IsdB	–	+	–	–	–	–	+	–	–	+	+
group_1846	Peptide-binding protein	cd08510: PBP2_Lactococcal_OppA_like; the substrate binding component of an ABC-type lactococcal OppA-like transport system	–	–	–	–	–	–	+	–	–	+	+
*group_669*	Hypothetical protein	Imm70 super family cl21402 Immunity protein 70	–	+	–	–	–	–	+	+	–	–	+
*group_1859*	DNA-binding response regulator	CitB COG2197 DNA-binding response regulator Similar to SalR of *S. suis* SC84 (Amino acid similarity, 86%; coverage, 100%)	–	+	–	–	–	–	+	+	–	–	+
*group_1860*	Histidine kinase	HisKA_3 super family cl26854 Histidine kinase Similar to SalK of *S. suis* SC84 (Amino acid similarity, 84%; coverage, 100%)	–	+	–	–	–	–	+	+	–	–	+
*group_1861*	ABC transporter permease	No conserved domain Blast hit with ABC transporter permeases of several species	–	+	–	–	–	–	+	+	–	–	+
*group_1862*	ABC transporter permease	No conserved domain Blast hit with ABC transporter permeases of several species	–	+	–	–	–	–	+	+	–	–	+
*group_1863*	ABC transporter	ABC_ATPase super family cl25403 ATP-binding cassette transporter nucleotide-binding domain	–	+	–	–	–	–	+	+	–	–	+
*group_1864*	ABC transporter	SunT super family cl26602 ABC-type bacteriocin/lantibiotic exporters	–	+	–	–	–	–	+	+	–	–	+
*group_391*	Lantibiotic-modifying protein	LanC_like super family cl04955 Cyclases involved in the biosynthesis of lantibiotics	–	+	–	–	–	–	+	+	–	–	+
*group_1865*	Columbicin A-like bacteriocin peptide	L_biotic_typeA super family cl04622 Type-A lantibiotic	–	+	–	–	–	–	+	+	–	–	+

^a^Based on pangenome analysis of 21 isolates using Roary (Additional file 9). Genes in a putative bacteriocin synthesis gene cluster are highlighted in italics.

^b^The presence or absence of the genes was investigated by TBLASTN using the gene products of DAT741 (coverage, > 80%; amino acid identities, > 80%).

### Variations were discovered in *cps* gene clusters of *S. ruminantium*

**Figure 2:**
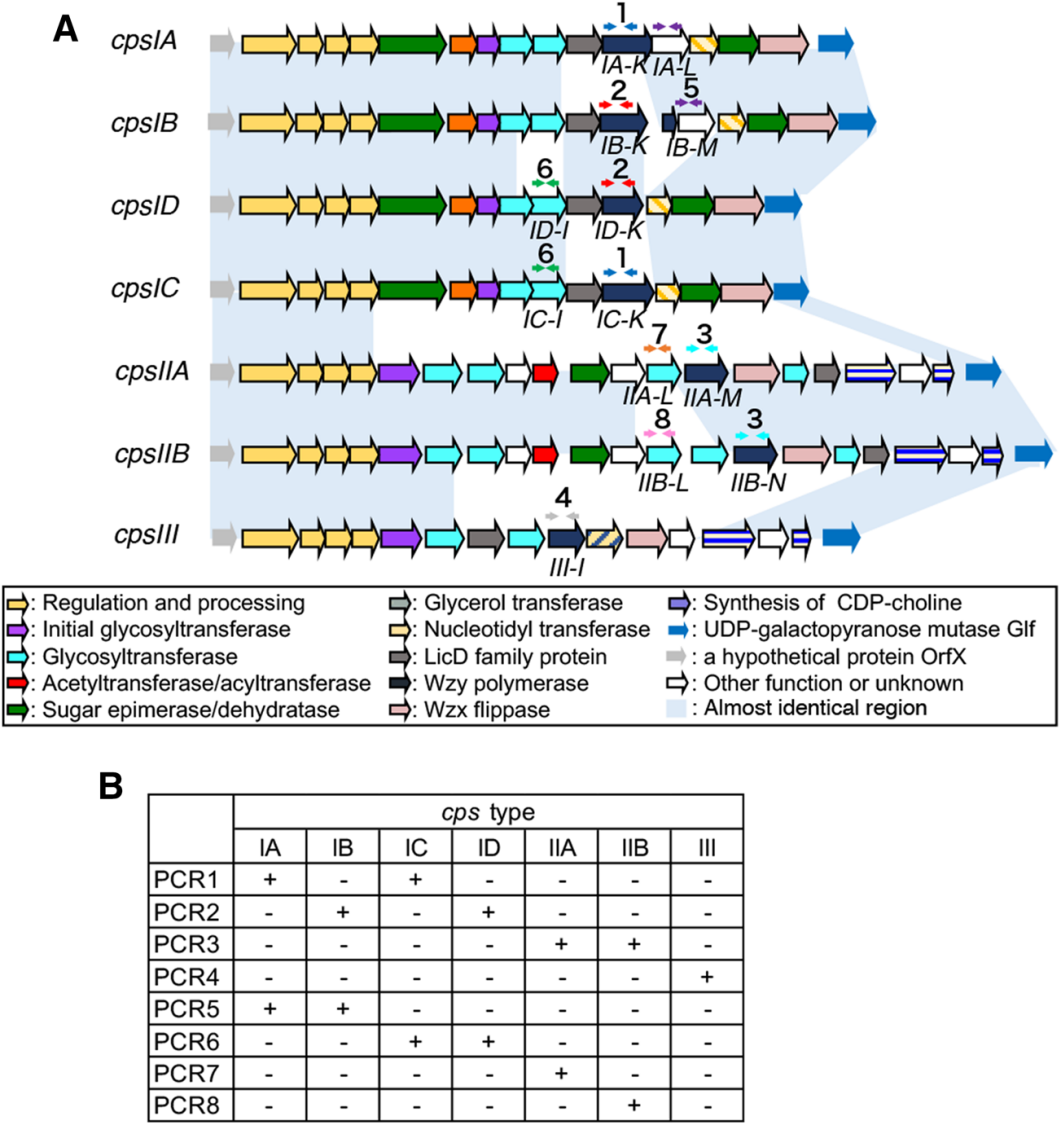
**Classification scheme for** ***S. ruminantium cps*** **gene clusters.** **A** Each coloured arrow represents a coding sequence, the colour of which indicates the predicted function. The target regions of the respective PCR assays are appended to the corresponding arrows. Light blue blocks indicate conserved regions according to pairwise BLASTN comparison data. **B** The *cps*-type classification scheme based on the PCR profiles.

In this study, *cps* type 33 isolates accounted for less than 25% of the tested isolates (Table 4 and Additional files 1 and 2). The genomes of all 20 *S. ruminantium* isolates exhibited putative *cps* genes between *orfX* and *glf* (Figure 2A and Additional file 10), and these genes were regarded as *cps* clusters of *S. ruminantium.* These *cps* clusters were divided into three main types (Type I–III) according to their genetic organization (Figure 2A and Additional file 10). Types I and II were further divided into four and two subtypes (Type IA–ID and IIA–IIB) based on the differences in one or two genes (Figure 2A). Among the sub-types, types IA and IC were classified into *cps* type 33 when analysed using the *cps*-typing scheme for *S. suis* [23]. In total, seven *cps* clusters (*cpsIA*-*D*, *cpsIIA*-*B*, and c*psIII*) were identified in the 20 isolates.

**Table 4:** Prevalence and distribution of *cps* types in 76 ruminant *S. ruminantium* isolates

*cps* type^a^	No. of isolates		
	Total, *n* = 76 (%)	Disease sites^c^, *n* = 34 (%)	Healthy animal and environment^d^, *n* = 23 (%)
IA (33^b^)	16 (21.1)	8 (23.5)	5 (21.7)
IB	20 (26.3)	9 (26.5)	7 (30.4)
IC (33^b^)	1 (1.3)	0	0
ID	1 (1.3)	0	1 (4.3)
IIA	11 (14.5)	5 (14.7)	3 (13.0)
IIB	10 (13.2)	5 (14.7)	3 (13.0)
III	2 (2.6)	2 (5.9)	0
UT	15 (19.7)	5 (14.7)	4 (17.4)

UT: untypable.

^a^Types based on the *S. ruminantium cps* gene cluster typing system developed in this study.

^b^*S. ruminantium cps* types IA and IC were classified as *S. suis cps* type 33 [23].

^c^Heart, lung, liver abscess, articular cavity or abscess in tympanic cavity (Table 1 and Additional file 1).

^d^Four of the 64 isolates collected by the diagnostic service of NIAH-Japan (Table 1 and Additional file 1) and 19 of the 22 *S. ruminantium* isolates collected from cattle tonsils (Additional file 2, three isolates were from a cow with mastitis or gastroenteritis).

To classify the seven identified *cps* clusters of *S. ruminantium* and investigate the distribution of the *cps* types in our isolates, we designed a PCR-based *cps*-typing scheme for *S. ruminantium* using eight primer pairs (Figure 2). Among the 76 *S. ruminantium* isolates used in this study, 61 isolates (80.3%) were classified into one of the seven types using this typing scheme, while 15 isolates (19.7%) were untypable (Table 4). The distribution of the *cps* types from the lesion-associated isolates (*n* = 34) was comparable to that of the isolates from healthy animals and the environment (Table 4), although isolates classified as Type III were only isolated from the lesions of diseased cattle (*n* = 2).

### Antibiotic resistance genes were located in ICE-like elements in *S. ruminantium*

**Figure 3:**
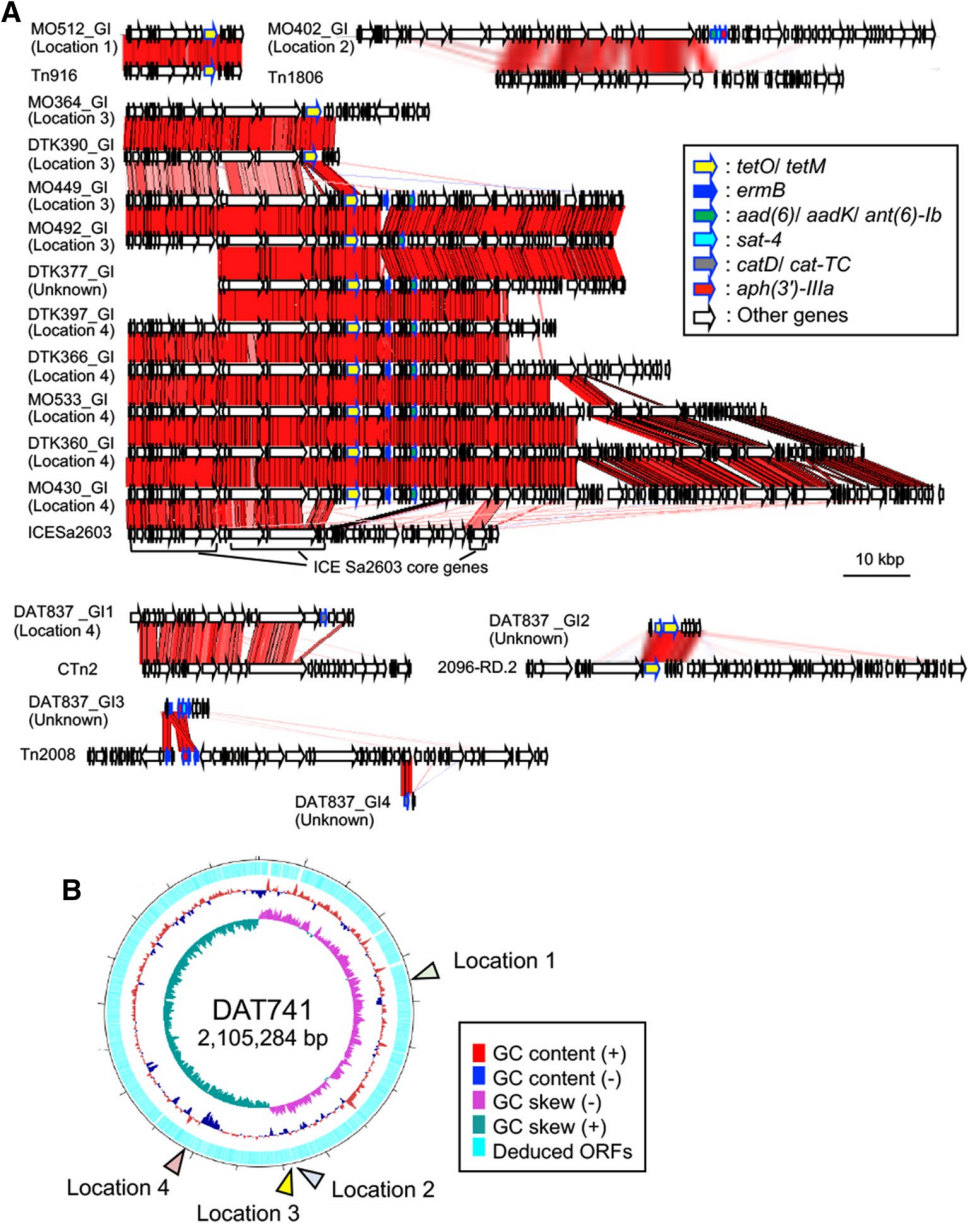
***S. ruminantium*** **GIs that carry antibiotic resistance genes (A) and their chromosomal locations (B).** **A** The GIs were compared with the representative ICEs Tn916, Tn1806, ICESa2603, Ctn2, Tn2008, and 2096-RD.2. Coloured arrows outlined by blue lines indicate antibiotic resistance genes; red blocks, conserved regions based on pairwise TBLASTX comparison data; brackets, core genes of ICESa2603a family ICEs. **B** Chromosomal locations of the GIs in the DAT741 genome. GC content, GC skew and deduced ORFs are represented in the inner map.

Our search of the 20 *S. ruminantium* genomes using CARD identified genes that putatively confer resistance to tetracyclines (*tetO* and *tetM*), aminoglycosides (*aph (3′)*-*IIIa*), streptomycin (*aad(6)*, *aadK* and *ant(6)*-*Ib*), phenicols (*catD* and *cat*-*TC*), macrolides (*ermB*), and streptothricin (*sat*-*4*) in 13 of the isolates (Table 5). Antibiotic susceptibility tests indicated remarkably high MIC values of tetracycline (≥ 16 μg/mL) in the isolates carrying *tetO* or *tetM,* with the exception of the *tetO*-positive strain DTK397 (MIC, 1.5 μg/mL) (Additional file 11). Furthermore, high MICs were noted for streptomycin (≥ 96 μg/mL) in the isolates carrying *aad(6)*, *aadK* or *ant(6)*-*Ib*; erythromycin (≥ 12 μg/mL) in the isolates carrying *ermB*; kanamycin (≥ 256 μg/mL) in the isolates carrying *aph (3′)*-*IIIa*; and chloramphenicol (24 μg/mL) in the isolate carrying *catD* and *cat*-*TC* (Additional file 11). Comparatively, the MICs were low for DAT741, which carried none of these genes (Additional file 11). Among the identified antibiotic resistance genes, *tetO, ermB* and *ant(6)*-*Ib* were present in more than 40% of the isolates, and ten isolates possessed multiple antibiotic resistance genes (Table 5). Further comparative analysis indicated that almost all of the resistance genes were located in genomic islands (GIs) at four different chromosomal loci (1–4; Figure 3 and Additional file 12). Notably, *tetO*, *sat*-*4, aad(6)*, *ermB*, *aph (3′)*-*IIIa* and *cat*-*TC* of DAT837 and *tetO*, *ant(6)*-*Ib* and *ermB* of DTK377 were the exceptions, as their chromosomal locations were not identified. The nucleotide sequences of these GIs showed similarities to those of ICEs present in the ICEberg database, particularly the ICESa2603 family (Figure 3A and Additional file 13).

**Table 5:** Antibiotic resistance genes found in 20 *S. ruminantium* genomes

Genes^b^	Isolation sites									
	Heart			Abscess			Lung			
	Chromosomal location of genomic islands carrying the antibiotic resistance genes^a^									
	–	Location 3	Location 3	–	–	–	Location 4	Location 4	Location 4	Location 4, UNK
	DAT741	DTK364	DTK390	DTK285	DTK284	MO591	DTK360	DTK397	DTK366	DAT837
*tetM*	–	–	–	–	–	–	–	–	–	–
*tetO*	–	+	+	–	–	–	+	+	+	+
*sat*-*4*	–	–	–	–	–	–	–	–	–	+
*aad (6)*	–	–	–	–	–	–	–	–	–	+
*aadK*	–	–	–	–	–	–	–	–	–	–
*ant(6)*-*Ib*	–	–	–	–	–	–	+	+	+	–
*ermB*	–	–	–	–	–	–	+	+	+	+
*aph(3′)*-*IIIa*	–	–	–	–	–	–	–	–	–	+
*catD*	–	–	–	–	–	–	–	–	–	+
*cat*-*TC*	–	–	–	–	–	–	–	–	–	+

Genes^b^	Isolation sites										Function	Confer resistance to
	Tonsils or oral cavity											
	Chromosomal location of genomic islands carrying the antibiotic resistance genes^a^											
	Location 2	UNK	Location 4	Location 3	Location 3	–	–	Location 1	Location 4	–		
	MO402	DTK377	MO430	MO449	MO492	MO498	MO505	MO512	MO533	MO557		
*tetM*	–	–	–	–	–	–	–	+	–	–	Tetracycline-resistant ribosomal protection protein	Tetracycline, doxycycline, minocycline, oxytetracycline, etc.
*tetO*	–	+	+	+	+	–	–	–	+	–		
*sat*-*4*	+	–	–	–	–	–	–	–	–	–	Streptothricin acetyltransferase	Streptothricin
*aad (6)*	–	–	–	–	–	–	–	–	–	–	Aminoglycoside nucleotidyltransferase	Streptomycin
*aadK*	+	–	–	–	–	–	–	–	–	–		
*ant(6)*-*Ib*	–	+	+	+	+	–	–	–	+	–		
*ermB*	–	+	+	+	–	–	–	–	+	–	23S ribosomal RNA methyltransferase	Erythromycin, clarithromycin, tylosin, vernamycin, etc.
*aph(3′)*-*IIIa*	+	–	–	–	–	–	–	–	–	–	Aminoglycoside phosphotransferase	Kanamycin, gentamicin, ribostamycin, butirosin, etc.
*catD*	–	–	–	–	–	–	–	–	–	–	Chloramphenicol acetyltransferase	Chloramphenicol, thiamphenicol, azidamfenicol
*cat*-*TC*	–	–	–	–	–	–	–	–	–	–		

UNK: unknown.

^a^Chromosomal location based on the complete genome of DAT741, which was sequenced in this study (Figure 3).

^b^Gene name determined using CARD analysis.

## Discussion

In the present study, many of the isolates biochemically identified as *S. suis* from diseased ruminants in Japan were re-identified as *S. ruminantium* according to the most recent classification*. S. suis* was also found to be involved in clinical cases in cattle with severe diseases or clinical signs such as astasia and meningitis. The isolation of *S. suis* from diseased ruminants has been reported not only in Japan but also in North America and Belgium [18–21], although it is uncertain whether these isolates were accurately speciated, as *S. ruminantium* had not yet been proposed. Notably, discrimination between *S. ruminantium* and *S. suis* through biochemical tests has traditionally been difficult. Indeed, at the onset of this study, there was no differentiating test available for diagnostic laboratories. Therefore, in the present study, we developed an *S. ruminantium*-specific PCR assay; this novel PCR assay, together with *recN*-PCR for *S. suis* detection, will be key to the accurate diagnosis of streptococcal diseases in ruminants.

This study also revealed that both *S. suis* and *S. ruminantium* can be isolated from the tonsils and oral cavities of healthy cattle. Notably, in a previous Belgian study, *S. suis* was isolated from the tonsils of healthy cattle in 40% of the investigated population [42]. Although these isolates were identified on the basis of biochemical tests, the prevalence was comparable to the isolation of *S. ruminantium* and *S. suis* reported in this study (42.7%). Thus, these findings suggest that *S. ruminantium* and *S. suis* can naturally inhabit the tonsils and oral cavity of cattle.

According to the MLST analysis, the *S. suis* clinical isolates from cattle were apparently different from the swine isolates and were typed as four novel STs. Considering that *S. suis* can be isolated from healthy cattle, *S. suis* is likely an opportunistic pathogen in cattle. In this study, no bovine *S. suis* isolates were grouped into CCs or serotype 2, which are frequently found in the isolates from patients with severe systemic diseases. However, a previous Japanese study reported a case of serotype 2 *S. suis* infection in a dairy farmer who had no history of contact with pigs [43]. Although serotype 2 *S. suis* was not detected in the cattle from this farm, serotype 2 *S. suis* has been isolated from diseased cattle in Canada and Belgium [18, 19]; therefore, bovine *S. suis* may cause human disease. Serotype 8 strains were most frequently isolated from healthy and diseased cattle in this study. Although it remains unclear why serotype 8 was isolated most frequently due to the limited data on ruminant isolates, our data suggest that the serotype 8 capsule may be one of the types of *S. suis* that could adapt to cattle in Japan. Further investigation by MLST and serotyping of *S. suis* isolates from diseased ruminants from geographically diverse regions will elucidate the presence or absence of CCs and serotypes strongly associated with ruminant disease and the risk of human disease posed by ruminant isolates.

The present study highlights that *S. ruminantium* may be associated with respiratory diseases, pneumonia, and torticollis in ruminants as well as endocarditis and arthritis, as previously reported [12, 17]. An earlier study also reported the association of *S. ruminantium* with various diseases in cattle [21], although the isolates were simultaneously identified as *S. suis*, showing the best 16S rRNA sequence matches with the *S. suis* serotype 33 type strain (99–100%). Collectively, the data suggest that *S. ruminantium* can be associated with various diseases in ruminants, although the associations observed in some cases, especially with respiratory diseases and pneumonia cases, are questionable due to the isolation of these bacteria from lesions together with other pathogens in the present and previous reports [21]. In our study, *S. suis* was more frequently isolated from the tonsils of healthy cattle than *S. ruminantium*, whereas *S. ruminantium* was more frequently found in the lesions of diseased ruminants than *S. suis* (Table 1). Therefore, *S*. *ruminantium* may be more likely to be associated with disease in ruminants than *S. suis*. However, PFGE analyses from this and previous studies [21] suggest that strains with various genetic backgrounds can be associated with diseases in ruminants. Furthermore, our PFGE data indicated that *S. ruminantium* genetic diversity was detectable at the farm level and that the pulsotype was not associated with health or disease status. Given that healthy cattle also carry *S. ruminantium*, *S. ruminantium* may contribute to the development of disease as a primary or secondary pathogen or may be a commensal or an opportunistic pathogen. Additionally, among the 11 COGs distinctively present in the clinical isolates identified by our analysis, two showed over 80% similarity to *salR* and *salK,* a two-component signal transduction system that contributes to the virulence of highly invasive *S. suis* 05ZYH33 in pigs [44]. Further analysis of the function of each distinctive COG, including these two, is necessary to elucidate whether these gene products contribute to virulence and disease severity.

In a previous study by Tohya et al. [12], all the *S. ruminantium* isolates obtained from cattle with endocarditis were classified as serotype 33. However, this study revealed variation in the *cps* gene clusters of *S. ruminantium* isolates. In addition, approximately 20% of the tested isolates were untypable by *cps*-typing PCR, possibly due to the existence of novel *cps* types that cannot be classified by PCR or have lost of some part of the *cps* gene cluster, including the target genes for PCR. Furthermore, the *cps*-typing PCR strategy for *S. ruminantium* described in this study only targets two genes, and thus, the possibility of the existence of more variations in the *cps* gene clusters cannot be ruled out. These data suggest that this species is serologically diverse, although additional data from the serological analysis of each *cps* type of isolate will be required. Our data implied no clear associations between *cps* type and ruminant disease. However, some isolates seemed to show high hydrophobicity when cultured in broth, implying that these isolates may be unencapsulated or produce low amounts of capsule. In *S. suis*, the presence of several unserotypable isolates that were typable by *cps* typing PCR has been reported [7, 23]; thus, further confirmation of capsule expression in isolates of each *cps* type will be necessary to clarify whether certain serological types of *S. ruminant* are associated with ruminant diseases.

A previous study [45] indicated that the *cps33* gene cluster shared relatively high amino acid sequence similarity with the *cps9* gene cluster. In fact, two-way cross-reactions between serotypes 9 and 33 have been reported [17]. According to our data, approximately 50% of *S. ruminantium* isolates were typed as Type IA–ID, which are identical (IA) or similar (IB–ID) to the *cps33* gene cluster. Furthermore, several serotype 9 *S. suis* isolates were found in cattle in previous studies [19, 20]. Although further serological analysis will be needed, this type of antigenicity may be preferable for adaptation to cattle. None of the *cps* gene clusters of known serotypes in *S. suis* showed amino acid sequence similarity in most of the region with the *cps* IIA–B and III gene clusters (data not shown).

In addition to variations in *cps* gene clusters, our genome analysis of 20 *S. ruminantium* isolates identified genes that putatively confer resistance to streptothricin, tetracyclines, macrolides, aminoglycosides, and phenicols, particularly in the isolates from the lungs, tonsils, and oral cavity, which are microenvironments that other bacterial species also inhabit. In *S. suis*, many antibiotic resistance genes have been shown to be located in ICEs [31, 46]. In *S. ruminantium*, many of the antibiotic resistance genes identified in this study were also located in ICESa2603 family ICEs, which are putative mobile genetic elements (MGEs) found in several *S. suis* and other streptococcal isolates from pigs and cattle in China [46, 47] (Figure 3 and Additional file 13). The transfer of some of the ICESa2603 family ICEs to other *Streptococcus* spp. has been experimentally verified [46, 47], and many of the ICE-like elements identified in this study included almost all of the core genes of this ICE family [46], as well as genes encoding the Type IV secretion system, integrase, and excisionase (Figure 3 and Additional file 12). Although further study of the transfer of the identified ICE-like elements is required, *S. ruminantium* may be a reservoir of antibiotic resistance genes that can transfer resistance genes to other streptococci via ICEs.

By contrast, beta-lactam resistance genes were not found in *S. ruminantium* in this study, and the MICs were low for these antibiotics (MIC range of amoxicillin, 0.012–0.08 µg/mL; ampicillin, ≤ 0.016 µg/mL; and benzylpenicillin, 0.016–0.023 µg/mL). Other groups have also reported high susceptibility to beta-lactams in *S. suis* and *S. ruminantium* [21, 48]. Although continuous studies are necessary to monitor antibiotic resistance in ruminant *S. suis* and *S. ruminantium*, these data suggest that penicillins are effective for the treatment of these infections in ruminants.

In conclusion, this study revealed that both *S. suis* and *S. ruminantium* can be associated with ruminant disease, and the differentiation between these two species is now aided by our novel PCR strategy, which will be key to the accurate diagnosis of streptococcal disease in ruminants. The information and tools established through this work will also contribute to the accumulation of *S. suis* and *S. ruminantium* data, resulting in an increased understanding of the epidemiology, pathogenicity, virulence, and zoonotic potential of these two species.

## Supplementary information

**Additional file 1** ***gdh*-PCR positive** ***Streptococcus*** **strains from ruminants in the collection of the National Institute of Animal Health.** Detailed information on the 64 strains listed in Table 1.

**Additional file 2. PCR positive** ***Streptococcus*** **strains collected from the tonsils of 110 cattle in this study.** Detailed information on the additional 55 strains from 110 cattle is listed in Table 2.

**Additional file 3.** ***Streptococcus*** **spp. strains tested by the** ***S. ruminantium*-specific PCR assay developed in this study.** Detailed information on the *Streptococcus* strains used for checking the specificity of the developed *S. ruminantium*-specific PCR.

**Additional file 4. Whole-genome-sequenced strains and general properties of the genomes.** Information on the genome sequence assembly of the 20 *S. ruminantium* isolates.

**Additional file 5. PCR primers for the typing of** ***cps*** **gene clusters in** ***S. ruminantium***. Information on the primer sequences, target genes, and product sizes under the developed *cps*-typing approach for *S. ruminantium.*

**Additional file 6. Alignment of partial regions of the 16S rRNA gene sequences of** ***S. suis*** **serotype reference strains and** ***S. ruminantium*** **GUT-187**^**T**^ **and DAT741.** Primer binding sites for the PCR assay developed in this study and their flanking regions are displayed (forward primer, *S. ruminantium* GUT-187^T^ positions 42–63; reverse primer, *S. ruminantium* GUT-187^T^ positions 269–290; accession no. LC195038). Red boxes represent primer binding sites. Yellow shaded letters indicate the deviations from the consensus found in the tested *S. ruminantium* strains. The letters highlighted with a grey background also indicate deviations from the consensus but were not found in the tested *S. ruminantium* strains.

**Additional file 7. Amplified products from the** ***S. ruminantium*-specific PCR assay with serial dilutions of an** ***S. ruminantium*** **DAT741 DNA template.** Lane 1, 2.7 × 10^7^ CFU/mL; 2, 2.7 × 10^6^ CFU/mL CFU/tube; 3, 2.7 × 10^5^ CFU/mL; 4, 2.7 × 10^4^ CFU/mL; 5, 2.7 × 10^3^ CFU/mL; 6, 2.7 × 10^2^ CFU/mL; 7, 27 CFU/mL; 8, Distilled water; M: molecular marker (100 bp + 3 k DNA Ladder, SMOBIO Technology, Taiwan).

**Additional file 8.** ***cps*** **types and sequence types (STs) of four** ***S. suis*** **clinical isolates from cattle used in this study.** Information on *cps* types and allele types determined by MLST of the 4 *S. suis* clinical isolates from cattle.

**Additional file 9. Presence/absence of genes of each COG among 21** ***S. ruminantium*** **strains.** All of the pangenome data of the 21 *S. ruminantium* strains analysed by Roary.

**Additional file 10. Presence/absence of genes of each COG related to capsular polysaccharide synthesis among 21** ***S. ruminantium*** **strains.** Detailed information on the *cps* genes identified from pangenome data of the 21 *S. ruminantium* strains

**Additional file 11. MICs of tetracycline, erythromycin, chloramphenicol, kanamycin and streptomycin in 14** ***S. ruminantium*** **strains.** MIC data of the 14 *S. ruminantium* isolates that carried antibiotic resistance genes.

**Additional file 12. Presence/absence of the genes of each COG in the genomic islands carrying antibiotic resistance genes identified by CARD analysis with 20** ***S. ruminantium*** **strains.** Detailed information on the locations and genomic islands that included the antibiotic resistance genes identified by CARD.

**Additional file 13. ICEs from the ICEberg database showing BLAST hits with the genomic islands carrying antibiotic resistance genes found in** ***S. ruminantium*** **isolates in this study.** First, three ICE hits with the genomic islands that included the identified antibiotic resistance genes.
