# Supplementary material for: Genotypic diversity of Streptococcus suis and the S. suis-like bacterium Streptococcus ruminantium in ruminants
Source: Vet Res. 2019 Nov 14;50:94. doi: 10.1186/s13567-019-0708-1 (PMC6854688; doi:10.1186/s13567-019-0708-1)
Supplement: Supplementary file 6 — Additional file 6. Alignment of partial regions of the 16S rRNA gene sequences of S. suis serotype reference strains and S. ruminantium GUT-187T and DAT741. Primer binding sites for the PCR assay developed in this study and their flanking regions are displayed (forward primer, S. ruminantium GUT-187T positions 42–63; reverse primer, S. ruminantium GUT-187T positions 269–290; accession no. LC195038). Red boxes represent primer binding sites. Yellow shaded letters indicate the deviations from the consensus found in the tested S. ruminantium strains. The letters highlighted with a grey background also indicate deviations from the consensus but were not found in the tested S. ruminantium strains. [file 13567_2019_708_MOESM6_ESM.pdf]

Forward primer

S. suis **(reference strains of serotypes 1-19,21,23-25,27-31,and 1/2)**

```
Other 27 serotypes   GCCTAATACATGCAAGTAGAACGC----TGAAGTCTGGTGCTT--GCACTA----GACGGATGAGTTGCG
Serotype 10          GCCTAATACATGCAAGTAGAACGC----TGAAGTCTGGTGCTT--TCACTA----GACGGATGAGTTGCG
Serotype 30          GCCTAATACATGCAAGTGGAACGCATGATGGATACCGGAGCTT--GCTCCACCATTCATCATGAGTCGCG
```

S. parasuis **(reference strains of serotypes 20,22,and 26)**

```
Serotypes 20 and 26  GCCTAATACATGCAAGTGGAACGCATGATTGATACCGGAGCTT--GCTCCACCATTAATCATGAGTCGCG
Serotype 22          GCCTAATACATGCAAGTGGAACGCATGATTGATACCGGAGCTT--GCTCCACCATTAATCATGAGTCGCG
```

S. orisratti **(reference strains of serotypes 32 and 34)**

```
serotype32           GCCTAATACATGCAAGTAGAACGC----TGAAGTCTGGTGCTT--GCACCG----GATGGATGAGTTGCG
serotype34           GCCTAATACATGCAAGTGGAACGCATGATTGATACCGGAGCTT--GCTCCATCATTAATCATGAGTCGCG
```

S. ruminantium **(reference strain of serotype 33 and two strains)**

```
serotype33           GCCTAATACATGCAAGTGGAACGCAACTTTTCACGCGTTTCTTCGGAAACATCTGAGAAGTTGAGTCGCG
DAT741               GCCTAATACATGCAAGTGGAACGCAACTTTTCATCCGTTTCTTCGGAAACATCTGAGAAGTTGAGTCGCG
GUT-187T             GCCTAATACATGCAAGTGGAACGCAACTTTTCATCCGTTTCTTCGGAAACATCTGAGAAGTTGAGTCGCG
                    31                                                                  100
```

Reverse primer

S. suis

```
14 serotypes         GGTAACGGCTCACCAAGGCTTCGATACATAGCCGACCTGAGAGGGTGATCGGCCACACTGGGACTGAGAC
15 serotypes         GGTAACGGCTCACCAAGGCATCGATACATAGCCGACCTGAGAGGGTGATCGGCCACACTGGGACTGAGAC
```

S. parasuis

```
3 serotypes          GGTAACGGCTCACCAAGGCTTCGATACATAGCCGACCTGAGAGGGTGATCGGCCACACTGGGACTGAGAC
```

S. orisratti

```
2 serotypes          GGTAAAGGCTCACCAAGGCGACGATACATAGCCGACCTGAGAGGGTGATCGGCCACACTGGGACTGAGAC
```

S. ruminantium

```
serotype33           GGTAAAGGCCTACCAAGGCAACGATACATAGCCGACCTGAGAGGGTGATCGGCCACACTGGGACTGAGAC
DAT741               GGTAAAGGCCTACCAAGGCAACGATACATAGCCGACCTGAGAGGGTGATCGGCCACACTGGGACTGAGAC
GUT-187T             GGTAAAGGCCTACCAAGGCAACGATACATAGCCGACCTGAGAGGGTGATCGGCCACACTGGGACTGAGAC
                    260                                                                 329
```
