# Supplementary figures and images for: Genotypic diversity of Streptococcus suis and the S. suis-like bacterium Streptococcus ruminantium in ruminants
Source: Vet Res. 2019 Nov 14;50:94. doi: 10.1186/s13567-019-0708-1 (PMC6854688; doi:10.1186/s13567-019-0708-1)

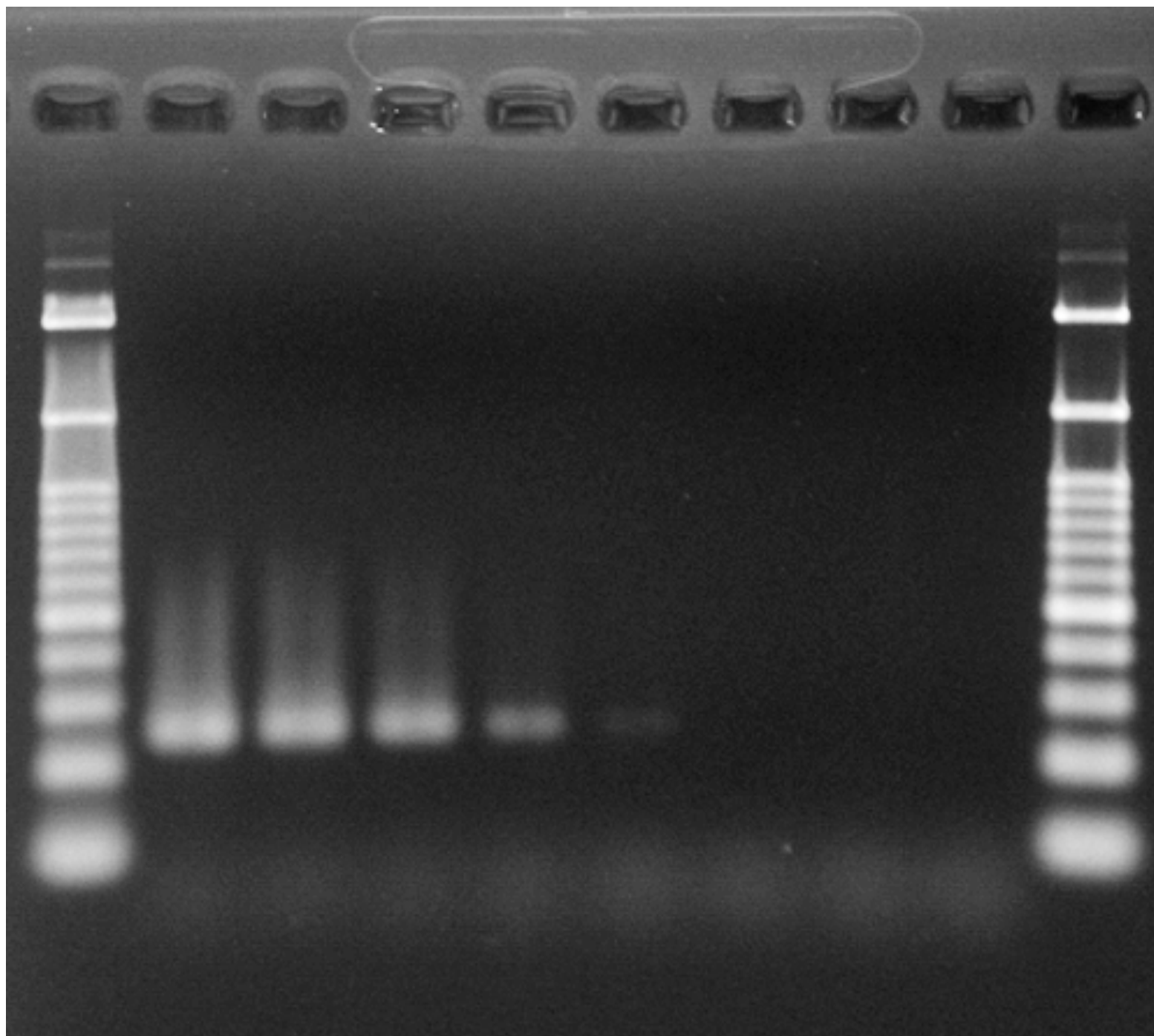

M 1 2 3 4 5 6 7 8 M

Supplement: Supplementary file 7 — Additional file 7. Amplified products from the S. ruminantium-specific PCR assay with serial dilutions of an S. ruminantium DAT741 DNA template. Lane 1, 2.7 × 10^7^ CFU/mL; 2, 2.7 × 10^6^ CFU/mL CFU/tube; 3, 2.7 × 10^5^ CFU/mL; 4, 2.7 × 10^4^ CFU/mL; 5, 2.7 × 10^3^ CFU/mL; 6, 2.7 × 10^2^ CFU/mL; 7, 27 CFU/mL; 8, Distilled water; M: molecular marker (100 bp + 3 k DNA Ladder, SMOBIO Technology, Taiwan). [file 13567_2019_708_MOESM7_ESM.pdf]
